# Effect of Nitrate on Nodule and Root Growth of Soybean (*Glycine max* (L.) Merr.)

**DOI:** 10.3390/ijms15034464

**Published:** 2014-03-13

**Authors:** Akinori Saito, Sayuri Tanabata, Takanari Tanabata, Seiya Tajima, Manabu Ueno, Shinji Ishikawa, Norikuni Ohtake, Kuni Sueyoshi, Takuji Ohyama

**Affiliations:** 1 Graduate School of Science and Technology, Niigata University, Niigata 950-2181, Japan; E-Mails: akinori1221@gmail.com (A.S.); sayuri.tanabata@gmail.com (S.T.); chukennoughtykoh@yahoo.co.jp (S.T.); manabu-u@adeka.co.jp (M.U.); ishikawa@agr.niigata-u.ac.jp (S.I.); ohtake@agr.niigata-u.ac.jp (N.O.); sueyoshi@agr.niigata-u.ac.jp (K.S.); 2 RIKEN Center for Sustainable Resource Science, Ibaraki 305-0074, Japan; E-Mail: takanari.tanabata@riken.jp

**Keywords:** soybean, nitrate, nodule, primary root, lateral root, light and dark conditions, 2D-PAGE, computer software

## Abstract

The application of combined nitrogen, especially nitrate, to soybean plants is known to strongly inhibit nodule formation, growth and nitrogen fixation. In the present study, we measured the effects of supplying 5 mM nitrate on the growth of nodules, primary root, and lateral roots under light at 28 °C or dark at 18 °C conditions. Photographs of the nodulated roots were periodically taken by a digital camera at 1-h intervals, and the size of the nodules was measured with newly developed computer software. Nodule growth was depressed approximately 7 h after the addition of nitrate under light conditions. The nodule growth rate under dark conditions was almost half that under light conditions, and nodule growth was further suppressed by the addition of 5 mM nitrate. Similar results were observed for the extending growth rate of the primary root as those for nodule growth supplied with 5 mM nitrate under light/dark conditions. In contrast, the growth of lateral roots was promoted by the addition of 5 mM nitrate. The 2D-PAGE profiles of nodule protein showed similar patterns between the 0 and 5 mM nitrate treatments, which suggested that metabolic integrity may be maintained with the 5 mM nitrate treatment. Further studies are required to confirm whether light or temperature condition may give the primary effect on the growth of nodules and roots.

## 1. Introduction

Soybean plants require a large amount of N because the seeds contain a high concentration of protein of approximately 35% to 40%, and the total amount of N assimilated in the shoot at harvest is proportional to the seed yield [1]. Soybean plants can form root nodules and use atmospheric N_2_ in association with rhizobia, nitrogen fixing soil bacteria. It is important to use both N_2_ fixation by the nodules and inorganic nitrogen assimilation by the roots to obtain the optimum yield of soybeans [1,2]. However, the development and nitrogen fixation activity of root nodules are known to be suppressed when nodulated roots are exposed to a high concentration of combined nitrogen. Nitrate, a major form of inorganic nitrogen in upland soil, strongly inhibits nodulation and N_2_ fixation activity [3–6]. Nitrate inhibition has been shown to have many effects, including a decrease in nodule number, nodule mass, and N_2_ fixation activity, as well as the acceleration of nodule senescence or disintegration; therefore, nitrate inhibition cannot be explained simply [5–9].

The nodulation of legume plants is systemically regulated by the previous infection called autoregulation of nodulation (AON) [10–12]. Several lines of hypernodulation or supernodulation mutants were isolated which lack AON and exhibited a profuse nodulation [13–15]. By a reciprocal shoot and roots grafting experiment of hypernodulation mutant line and wild type, it was found that AON is controlled by the shoot and not by roots [14]. All the hypernodulation mutants showed tolerance to nitrate for the nodule formation, and it is considered that there are common mechanism between nitrate inhibition of nodulation and AON [14–17]. Genes responsible for regulation of nodule number have been identified such as *LjHAR1* in model legume *Lotus japonicus* and *GmNARK* in soybean (reviewed in [12]). These genes encode leucine-rich repeat receptor kinases similar to *CLAVATA1* in *Arabidopsis thaliana* which controls shoot meristem development. Small peptide named CLE peptides are postulated as a candidate of the infection signal from roots to shoot, based on the similarity of CLAVATA1 and CLE peptide signaling [12,17–19]. Recently nitrate inducible CLE peptides have been found in addition to rhizobial infection induced CLE peptides [17,18]. It is postulated that inhibitor is synthesized in the shoot when NARK protein receive CLE peptides. It is transported from shoot to roots, and it prevents differentiation of nodules. However, growth of nodules already differentiated is not prevented by AON. Therefore, that mechanism of inhibition of nodule growth by nitrate might be different from that of nodule formation by nitrate and AON. Intensive research has been done for the regulation of nodule number of leguminous plants; however, much less attention has been paid to the mechanisms and the environmental effects on the nodule growth.

The inhibition of nodule growth in soybeans by nitrate is also complex. For example, the effects of nitrate on nodule growth were either negative or positive depending on the nitrate concentrations, placement, growth medium, and the treatment period [5,20–23]. Previous studies reported that nitrate inhibition was primarily host-plant-dependent and was independent of nitrate metabolism in rhizobia [3,24]. Many hypotheses have been proposed for the cause of nitrate inhibition of nodulation and N_2_ fixation activity, *i.e.*, carbohydrate-deprivation in nodules [6,25–27], feedback inhibition by a product of nitrate metabolism such as glutamine [28] or asparagine [29], and decreased O_2_ diffusion into the nodules, which restricts respiration of bacteroids, a symbiotic state of rhizobia [27,30,31].

We previously investigated the effects of nitrate on the nodule growth of soybean plants using hydroponic cultivation, and the diameters of the horizontal axis of individual nodules were measured using a slide caliper [32], Nodule growth and N_2_ fixation activity were shown to be rapidly depressed by the addition of 5 mM nitrate in the solution within a few days, whereas the inhibition of nodule growth and N_2_ fixation activity by nitrate was reversible by changing 5 mM nitrate in the culture solution to 0 mM nitrate. The nodule growth was monitored using a computer microscope and diameters of the horizontal axis of individual nodules were measured once a day [25], the diameters of root nodules increased from 1 to 6 mm for 14 days under 0 mM nitrate conditions. On the other hand, the increase in nodule diameter was almost completely stopped by the addition of 5 mM nitrate after the second day of the treatment.

Tanabata *et al*. [33] recently developed new computer software to measure nodule areas by fitting the circumference of a nodule to the oval line such that the area can be calculated from the major and minor axis of the oval line. We took close-up photographs of the nodulated roots of soybean using a digital camera in a photochamber every 1 h; and these photographs were subsequently used to measure the nodule area. The nodule area measured by this method was highly correlated with nodule dry weight (*R* = 0.959) [33]. The difference in growth rate of nodules was not observed for 48 h under continuous dark conditions with or without periodical flash at 1 h intervals [33]. This method is non-invasive to the nodules and roots, and growth can be more precisely determined than with slide calipers or a computer microscope [33]. In this study, we observed the short-term growth rate of soybean nodules for 16 h with 0 or 5 mM nitrate, which had been grown under light at 28 °C for 16 h and dark at 18 °C for 8 h conditions, in which hydroponic soybean plants grow well. We also monitored the growth rate of the primary root and lateral roots under the same treatment conditions to compare the effects of the treatments on nodule growth.

Soybean seed grow a primary root from the embryo and lateral roots from the primary root. The growth of lateral roots have been shown to be affected by environmental conditions, such as water and nutrient availability. Drew [34] reported that the growth of lateral roots was locally enhanced in a soil compartment with a high concentration of phosphate, nitrate, or ammonium, although they did not respond to potassium. In soybeans, the treatment with 0 mM nitrate promoted the elongation of the primary root, whereas 5 mM nitrate increased lateral root growth [22]. The mechanisms of the initiation of lateral roots have been studied previously [35,36]; however, the regulation of primary and lateral root growth in relation to nodule growth in soybeans has not yet been fully evaluated.

In the present study, we developed a rapid and non-invasive measurement system of soybean nodule growth and applied it to examine the effects of light/dark and 0 mM/5 mM nitrate treatments. The effects of these treatments on the primary root and lateral roots were also determined. Furthermore, we analyzed nodule proteins using 2D polyacrylamide gel electrophoresis (2D-PAGE) to investigate the effects of nitrate on the integrity of nodule metabolism.

## 2. Results and Discussion

### 2.1. Effect of 5 mM Nitrate Supply in the Culture Solution on Nodule Growth under Light and Dark Conditions

**Figure 1.**
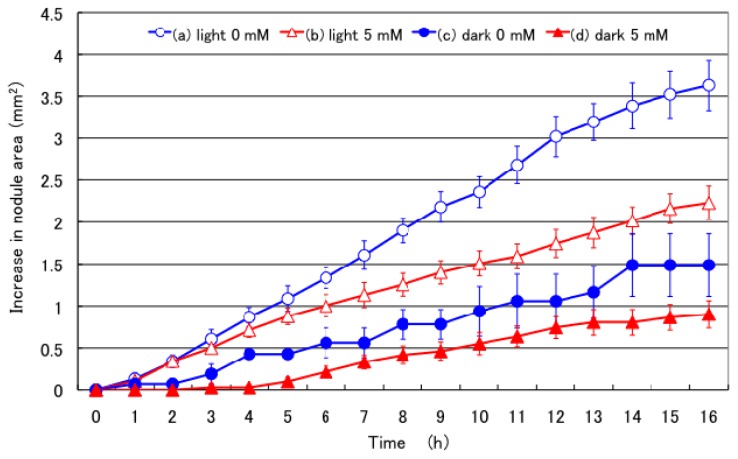
Effects of supplying 5 mM nitrate to the culture solution on soybean nodule growth under light or dark conditions. (a) Light 0 mM: Plants were cultivated with N free culture solution under light conditions at 28 °C; (b) Light 5 mM: Plants were cultivated with culture solution containing 5 mM nitrate under light conditions at 28 °C; (c) Dark 0 mM: Plants were cultivated with N free culture solution under dark conditions at 18 °C; (d) Dark 5 mM: Plants were cultivated with culture solution containing 5 mM nitrate under dark conditions at 18 °C. The 5 mM nitrate treatment (5 mM sodium nitrate was added to the culture solution), and 0 mM nitrate treatment were imposed from time 0. The graph shows the increase in the area of the oval figure fitted to the nodule figure by a computer program. Each data point shows the average and standard error of eleven replications of each treatment. The nodule growth was statistically different (*p* < 0.01) compared with light 0 mM treatment from 8 h in light 5 mM, from 4 h in dark 0 mM, and from 2 h in dark 5 mM treatment by *T*-test.

Figure 1 shows the effects of supplying 5 mM nitrate in the culture solution on the nodule growth of soybeans cultivated in a glass container. The graph shows the increase in the area of the oval figure fitted to the circumference of a nodule figure by the computer program [33] (This program is open access in the URL: http://phenotyping.image.coocan.jp/nodame/ [37]). The nodule area of the plant with the 0 mM nitrate treatment under light conditions consistently increased, although the rate of this increase was slower during the initial 2 h. The increase in the nodule area at 16 h was 3.6 mm^2^, which was the highest among the different treatments examined. This result indicated that the nodule growth rate in young soybean nodules was relatively constant during the daytime irrespective of the time of a day. The initial delay in nodule growth may have been due to the time-lag in photoassimilate transported from the shoot to nodules. The nodule area of the plant treated with 5 mM nitrate under light conditions showed a similar growth to the plant treated with 0 mM nitrate until the initial 2 h, and became significantly (*p* < 0.05) slower after 7 h. The final increase in the nodule area at 16 h after the nitrate treatment was 2.2 mm^2^, and approximately 60% of an increase in the nodule area with 0 mM nitrate under light conditions. The nodule area of the plant treated with 0 mM nitrate under dark conditions showed a low, but constant increase after an initial time-lag of approximately 2 h. The increase in nodule area was significantly (*p* < 0.05) lower than that with light 0 mM nitrate immediately after beginning of treatment, and the final increase in nodule area at 16 h was 1.5 mm^2^ and 42% of the nodule size with 0 mM nitrate under light conditions. The nodule area of the plant treated with 5 mM nitrate under dark conditions showed the lowest rate of growth among the treatments examined. The growth rate showed an initial 4 h time-lag, followed by a constant increase. The final increase in the nodule area at 16 h was 0.9 mm^2^, 25% that of the light 0 mM nodules.

This is the first study to measure short-term non-invasive nodule growth in intact soybean plants. Nodule growth under light conditions started just after the plant was changed from night to day conditions. Nodule growth rapidly responded to the day conditions because the treatments were performed just after an 8-h night period. The nodule growth under light conditions quickly started at 1 or 2 h. Fujikake *et al*. [25] reported that when ^11^CO_2_ positron emitting radioisotope was exposed to the soybean shoot, ^11^C reached the upper part of nodules and roots in 40 min, as observed using a positron emitting tracer imaging system. Ito *et al.* [38,39] reported that an appreciable amount of ^14^C was allocated to the underground part of young soybeans within 2 h of ^14^CO_2_ exposure to the shoot. On the other hand, nodules grown under dark conditions could grow slowly after 2 h (0 mM nitrate) or 4 h (5 mM nitrate) time-lag. The depletion of photoassimilates in the night might be an essential factor that reduced nodule growth. The 5 mM nitrate treatment depressed nodule growth under both light and dark conditions. Previous findings of daily changes in nodule diameter [25] showed that nodule growth continued a little for one day after starting the 5 mM nitrate treatment, and completely stopped after 2 days. Plants can use reserve carbohydrate, such as sugar or starch, for at least one day in addition to current photoassimilates. The results presented in this paper indicate that nitrate inhibition occurred very quickly several hours after the addition of 5 mM nitrate, and the inhibitory effect by nitrate and darkness were additive. In this experiment, light/dark treatments were imposed at different temperatures at 28 and 18 °C, respectively. Further studies are required whether light or temperature condition may give primary effect on the growth of nodules and roots.

### 2.2. Effect of Nitrate Supply to the Culture Solution on the Growth of the Primary Root under Light and Dark Conditions

**Figure 2.**
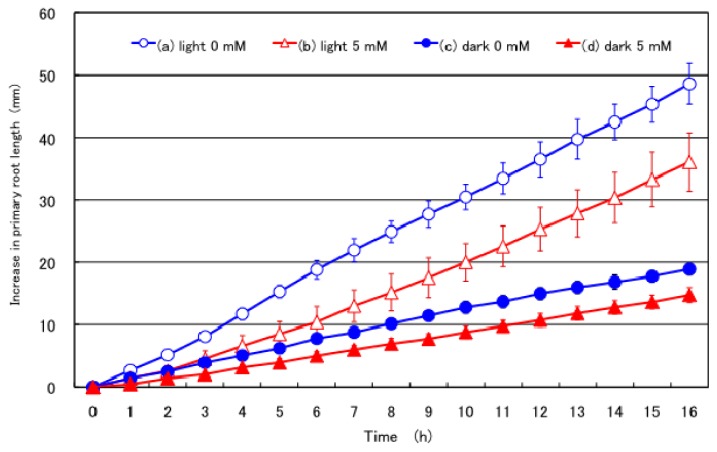
Effects of supplying 5 mM nitrate to the culture solution on the growth of the primary root under light or dark conditions. (a) Light 0 mM: Plants were cultivated with N free culture solution under light conditions at 28 °C; (b) Light 5 mM: Plants were cultivated in the culture solution with 5 mM nitrate under light conditions at 28 °C; (c) Dark 0 mM: Plants were cultivated with N free culture solution under dark conditions at 18 °C; (d) Dark 5 mM: Plants were cultivated in the culture solution with 5 mM nitrate under dark conditions at 18 °C. The graph shows increases in the length of the primary root. Each data point shows the average and standard error of four replications of each treatment. The primary root growth was statistically different (*p* < 0.01) compared with light 0 mM treatment by *T*-test from 2 h in dark 0 mM, and from 2 h in dark 5 mM treatment. Statistical difference (*p* < 0.05) was observed from 5 to 8 h in light 5 mM nitrate treatment.

Figure 2 shows the effects of supplying nitrate to the culture solution on the growth of the primary root under light or dark conditions. The primary root length of the plant with 0 mM nitrate treatment under light conditions consistently increased without any time-lag. The increase in the length of primary root at 16 h was 48 mm, which was the highest observed among the treatments examined. This result indicated that the growth rate of the primary root in young soybeans was constant during the daytime irrespective of the time of day. The growth rate of the primary root of the plants with the 5 mM nitrate treatment under light conditions initially showed the slow growth rate, but then increased to the same level as the 0 mM nitrate treatment under light conditions. The final increase in the length of the primary root at the 16 h treatment period was 35 mm, and approximately 73% of the length increase observed in 0 mM plants under light conditions. The growth of the primary root of the plants with the 0 mM nitrate treatment under dark conditions was significantly slower from 1 h after treatment (*p* < 0.05), but constant without any initial time-lag. The final length of the primary root at 16 h was 19 mm and 40% that of the 0 mM primary root under light conditions. The final increase in the primary root length of plants treated with 5 mM nitrate under dark conditions showed the lowest among the treatments examined without a time-lag. The final increase in the primary root length at 16 h was 14 mm, and 29% that of the 0 mM treatment under light conditions.

The effects on primary root growth were similar to those on nodule growth, except that no time-lag was observed. The growth rate of the primary root was faster with the light 0 mM treatment, light 5 mM nitrate, dark 0 mM nitrate, and dark 5 mM nitrate, in this order. When inhibition rates standardized to the light 0 mM treatment as 100% were compared, the light 5 mM nitrate treatment showed 60% nodule growth and 73% primary root length, dark 0 mM nitrate treatment showed 42% nodule growth and 40% primary root length, and dark 5 mM nitrate treatment showed 25% nodule growth and 29% primary root length. These results indicated that a common mechanism might be responsible for the inhibition of nodule growth and primary root growth by nitrate.

### 2.3. Effect of Nitrate Supply to the Culture Solution on the Growth of Lateral Roots under Light and Dark Conditions

**Figure 3.**
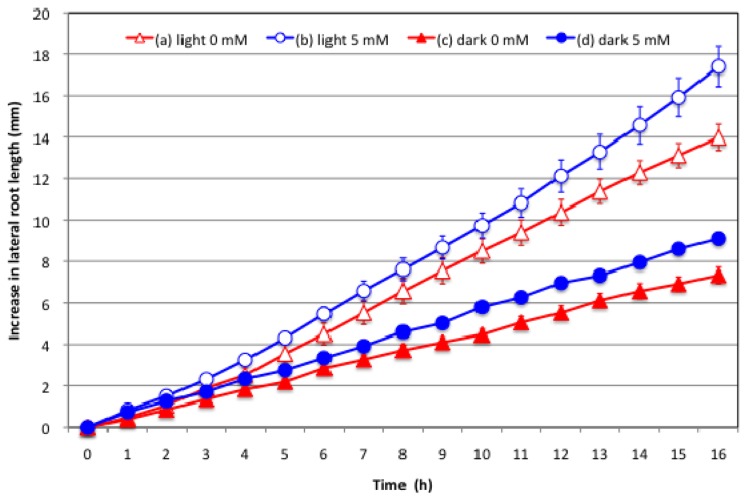
Effects of supplying nitrate to the culture solution on soybean lateral root growth under light or dark conditions. (a) Light 0 mM: Plants were cultivated in N free culture solution under light conditions at 28 °C; (b) Light 5 mM: Plants were cultivated in the culture solution with 5 mM nitrate under light conditions at 28 °C; (c) Dark 0 mM: Plants were cultivated in N free culture solution under dark conditions at 18 °C; (d) Dark 5 mM: Plants were cultivated in the culture solution with 5 mM nitrate under dark conditions at 18 °C. The graph shows the increase in the length of the lateral root. Each data point shows the average and standard error of eight replications of lateral root growth in each treatment. The lateral root growth was statistically different (*p* < 0.01) compared with light 0 mM treatment from 5 h in dark 0 mM, and from 8 h in dark 5 mM treatment by *T*-test. Statistic difference (*p* < 0.05) was observed from 15 h in light 5 mM nitrate treatment.

Figure 3 shows the effects of supplying nitrate to the culture solution on lateral root growth under light or dark conditions. Increases in the length of the lateral root of the plant with the 0 mM nitrate treatment under light conditions occurred in two phases: a consistent increase after 4 h with an initial slow increase during 0–4 h. The increase in the length of lateral roots at 16 h was 14 mm, which was the second highest observed among the treatments examined. The growth rate of the lateral roots of the plant with the light 5 mM nitrate treatment was slow during the initial 4 h, then increased more rapidly than that of light 0 mM nitrate plants. The final increase in the lateral root length at 16 h after the treatment was 17 mm, and approximately 121% of that of 0 mM plants under light conditions with significant differences (*p* < 0.05). The lateral root growth of the plant with the 0 mM nitrate treatment under dark conditions showed significantly slower after 4 h, but constant increase without any initial time-lag. The final increase in the lateral root length at 16 h was 7.2 mm, and 51% that of 0 mM lateral roots under light conditions. The final increase in the lateral root length of the plants treated with 5 mM nitrate under dark conditions was higher than that of 0 mM nitrate under dark conditions without a time-lag. The final increase in the lateral root length at 16 h was 9.0 mm, 64% that of the 0 mM primary root under light conditions.

The effect of supplying 5 mM nitrate on lateral root growth was different from that on primary root growth. The addition of 5 mM nitrate promoted lateral root growth under both light and dark conditions, although it depressed the growth of the primary root and nodules. A long-term cultivation with the 5 mM nitrate treatment produced a shorter root system, but larger number of lateral roots than that with the 0 mM nitrate treatment [23]. The response of the lateral roots to nitrate in this study was very rapidly started in several hours after the treatments. The effect of depressing lateral root growth under dark conditions was similar to that in the primary root and nodules. Therefore, the transport of current photoassimilates is primarily a regulating factor for all underground parts: nodules, the primary root, and the lateral roots.

### 2.4. Effect of Supplying Nitrate to the Culture Solution on Nodule Protein

**Figure 4.**
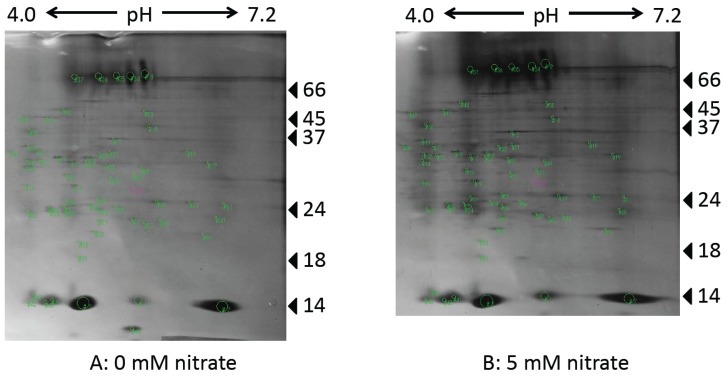
2D-PAGE of soybean nodules with the 0 and 5 mM nitrate treatments. (**A**) Plant cultivated with 0 mM nitrate; (**B**) Plant cultivated with 5 mM nitrate.

Figure 4 shows the protein profiles of soybean nodules by 2D-PAGE from the plants at 34 days after planting (DAP) treated with 0 mM (A) or 5 mM (B) nitrate for 24 days from 10 DAP. The strong spots on the lower part of the gel were components of leghemoglobin a (Lba) (spot #2) and Lbc (spot #3) [40]. Many protein spots were observed and the pattern of the protein spots were essentially similar between the 0 and 5 mM nitrate treatments; however, the brightness of the spots of the nodule-specific proteins, Lba and Lbc (the sum of Lbc_1_, Lbc_2_, Lbc_3_) were lower with the 5 mM nitrate treatment than with the 0 mM nitrate treatment. Therefore, the function of these nodules may be maintained even though nodule growth was arrested.

Lb is a red myoglobin-like hemoprotein specifically present in legume nodules [41]. Lb has a high affinity for binding oxygen (O_2_) and plays an essential role in N_2_ fixation in root nodules by maintaining O_2_ concentrations in infected area [42]. Four major Lb components have been identified in soybean nodules: Lba, Lbc_1_, Lbc_2_, and Lbc_3_ [41]. Nishiwaki *et al*. [43] and Sato *et al*. [44] determined the concentration of the leghemoglobin components, Lba, Lbc_1_, Lbc_2_, and Lbc_3_, and showed that these concentrations were lower in nodules supplied with 5 mM nitrate than in those supplied with 0 mM nitrate. The similar result was obtained in pea root nodules supplied with ammonium nitrate [45]. The results of 2D-PAGE in this study also showed that nitrate depressed the accumulation of Lb proteins.

The effects of nitrate may be due to either changes in nodule metabolism, changes in root metabolism, or both. The long-term effects of nitrate on the local and systemic growth of nodules and roots were investigated in an analysis of the vertical separating root system using the two-layered pot system [22,23]. Both the local and systemic inhibition of nodule growth (DW) and nitrogen fixation activity (Acetylene reduction activity) were observed with continuous supply of 5 mM nitrate [22]. However, supplying 1 mM nitrate from the lower pot promoted nodule growth and nitrogen fixation activity in the upper pot [23].

### 2.5. A Model for the Effects of a Short-Term Supply of Nitrate on the Growth of Nodules, Primary Roots, and Lateral Roots under Day/Night Conditions

Although it has been previously suggested that supplying nitrate has complex and multiple effects on nodule growth and nitrogen fixation activity, the quick and reversible inhibition of soybean root nodule growth and nitrogen fixation was found to involve a decrease in the supply of photoassimilates to the nodules [25]. These results indicated that the decrease in the supply of photoassimilates to nodules may be involved in the quick and reversible nitrate inhibition of soybean nodule growth and nitrogen fixation. However, when and how nitrate supply inhibits nodule growth in the short-term remains unknown because nodule growth was measured at day-intervals in the previous report [25].

As shown in Figure 1, the growth of nodules after plants were changed from the 8-h dark period to light conditions in 0 mM nitrate solution quickly started and continued during the 16 h of day time. The addition of 5 mM nitrate inhibited nodule growth shortly after 7 h of the light period, and significantly inhibited growth at the end of the 16-h light period. On the other hand, nodule growth under continuing dark conditions stopped immediately after starting treatment, and then grew slowly. Growth may depend on the carbohydrates stored in nodules or other plant parts, although it is possible that only water intake may induce expansion of the cells and promote the growth of roots and nodules. An additive inhibitory effect was observed under dark and 5 mM nitrate conditions. This result demonstrated that nitrate inhibition starts very quickly several hours after the addition of nitrate.

The effects of 5 mM nitrate on the increase in the primary root length under light or dark conditions were similar to those observed on nodule growth (Figures 1 and 2), although it was different from those on the lateral roots (Figure 3). The growth of the primary root and lateral roots was less under dark conditions than under light conditions; however, the addition of 5 mM nitrate inhibited the growth of the primary root, but promoted lateral root growth under both light and dark conditions.

**Figure 5.**
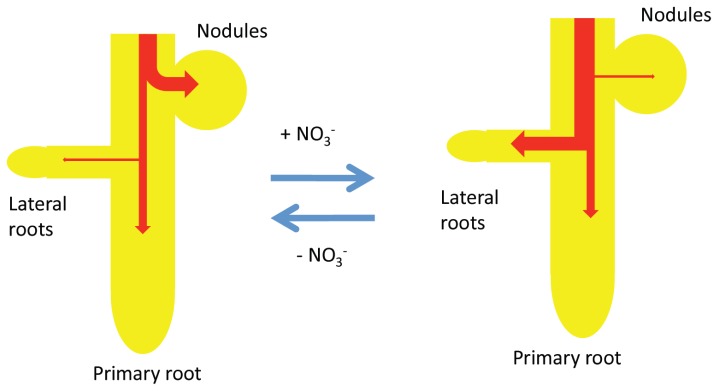
A model for the effects of nitrate on the growth of nodules, the primary root, and lateral roots.

A model for the effects of nitrate on the growth of root nodules, primary root, and lateral roots is proposed based on the results obtained here and previous findings (Figure 5). Under N free conditions in which soybean plants depend totally on nitrogen fixation, a large amount of the photoassimilate supplied to the underground part is used for nodule growth, nitrogen fixation, and assimilation processes. A previous study [25] investigated the partitioning of ^14^C photoassimilate in soybean plants at 22 DAP after 1 day of a 0 or 5 mM nitrate treatment. Distribution of ^14^C photoassimilate to the nodule was decreased by addition of 5 mM nitrate to hydroponic solution, instead, distribution of ^14^C to roots was increased. In addition, the increase in ^14^C in roots was mainly in the lateral roots supplied with nitrate. The results of measuring the growth of lateral roots (Figure 3) are consistent with the former results.

The number and placement of lateral roots was not predetermined, but was markedly affected by the availability of water and nutrients in the soil [36]. A lateral root is initiated from the pericycle cells immediately adjacent to the xylem poles. The phytohormone, auxin, is involved in the initiation process of lateral roots. After initiation, cells divide and expand to produce a lateral root primordium, which then breaks through the cell layers of the parent root. Finally, lateral root growth occurs by cell divisions in the root apical meristem [36]. The growth of lateral roots and primary roots are similar after a root emergency. Cell division in the apical meristem provides the cells and differentiates to various parts of the matured roots in the elongation (extension) zone 1 mm from the root tip. Therefore, plant roots elongate by cell division, differentiation, and cell expansion. Root growth is primarily supported by the photoassimilate supplied from shoot, although some other external and internal factors may also be involved.

According to a review of Sprent and James [46], the date of origin of legumes may be 59 million years ago, and two types of nodule development appear to have been established at approximately the same time. One is that symbiotic bacteria penetrate into the root through an infection thread from the root hair, and the other is a crack infection in which rhizobia enter from cracks in the roots made by lateral root growth. Moreover, determinate and indeterminate types have evolved among legume species. The indeterminate type of nodule is cylindrical in shape and has the meristem on the top, with a subsequent infection zone by rhizobia, then the basal part is the senescent zone. The determinate type of nodules like soybean is globular in shape, do not have a meristem, and differentiation is completed during the early stages, in which the nodule size is smaller than 1 mm. Therefore, nodule growth appears to have been caused by cell expansion and not cell proliferation.

In the case of soybeans, the underground part has nodules, a primary root, and lateral roots. The regulation of carbohydrate supply to the appropriate organs is fitted to the nutritional demand of nitrogen. When N availability is low in the soil, the plant preferentially supports nodule growth and nitrogen fixation activity. On the other hand, when nitrate levels are high in the soil, the plant promotes lateral root growth to efficiently absorb nitrate. The higher consumption of photoassimilate in roots may reduce the supply of photoassimilate to the nodules, and subsequently arrest nodule growth and nitrogen fixation activity. However, when nitrate is removed from the culture medium, the plant once again supplies carbohydrates to the nodules [23]. Further studies are needed to investigate the molecular mechanisms underlying the rapid inhibitory effects of nitrate on nodule growth and nitrogen fixation. In addition, soybean plants in this experiment were cultivated under light at 28 °C and dark at 18 °C, so it remains unclear whether light or temperature might affect the nodule and root growth rate. The formation of nodules may affect the growth of primary root and lateral roots in soybean plants. The analysis of the growth of primary and lateral roots of non-inoculated soybeans or non-nodulating lines may be important to investigate the effect of nodule formation on the root growth.

## 3. Experimental Section

### 3.1. Plant Material and Growth Conditions

Soybean seeds (*Glycine max* (L.) Merr., cultivar “Williams”) were sterilized with 70% ethanol for 30 s and sodium hypochlorite solution (0.5% Cl), and were washed thoroughly with water. Seeds were then inoculated with a suspension of *Bradyrhizobium japonicum* (strain “USDA110”) at concentration of 10^8^ cells·mL^−1^ just after 7 days incubation in YM liquid medium at 28 °C and sown in a vermiculite tray. Six days after planting (DAP), each plant was transplanted to a glass container box (12 cm × 8 cm, 10 cm height) with 800 mL of nitrogen free nutrient solution [11] with K_2_SO_4_ 109 g·m^−3^, KCl 0.935 g·m^−3^, K_2_HPO_4_ 8.5 g·m^−3^, CaCl_2_·2H_2_O 183 g·m^−3^, MgSO_4_·7H_2_O 123 g·m^−3^, H_3_BO_4_ 0.367 g·m^−3^, CuSO_4_·5H_2_O 0.032 g·m^−3^, MnSO_4_ 0.189 g·m^−3^, ZnSO_4_·7H_2_O 0.144 g·m^−3^, (NH_4_)_6_Mo_7_O_24_ 0.004 g·m^−3^, CoSO_4_ 0.028 g·m^−3^, NiSO_4_·6H_2_O 0.0035 g·m^−3^, EDTA·2Na 18.6 g·m^−3^, FeSO_4_·7H_2_O 13.9 g·m^−3^, pH at 5.8–6.0. The culture solution was continuously aerated by an air pump, and changed three times a week. Seeds were cultivated in a photochamber (MLR-350, Sanyo, Japan) 28 °C/18 °C day/night temperature, 55% relative humidity, 228 mmol·m^−2^·s^−1^ PPFD, 16-h photoperiod and 8-h dark period.

### 3.2. Monitoring Nodule Growth by the Addition of 5 mM Nitrate under Light or Dark Conditions

Plants were separated into two chambers with different light and temperature conditions at 13 DAP; light (28 °C) or dark (18 °C) just after the end of the 8-h dark period. Sodium nitrate was added to N free solution to prepare the culture solution with 5 mM nitrate. Photographs of the nodulated roots in the glass container were taken by a digital camera (COOL PIX S10, Nikon, Tokyo, Japan) with a high resolution (2816 × 2112 pixels) at 1-h intervals. We used a flashlight when the photograph was taken under dark conditions and covered the container and shaded the camera with aluminum foil. It was confirmed that the use of the flashlight had no significant effect on nodule growth under the same conditions [33].

### 3.3. Calculation of Nodule Growth by a Computer Program

**Figure 6.**
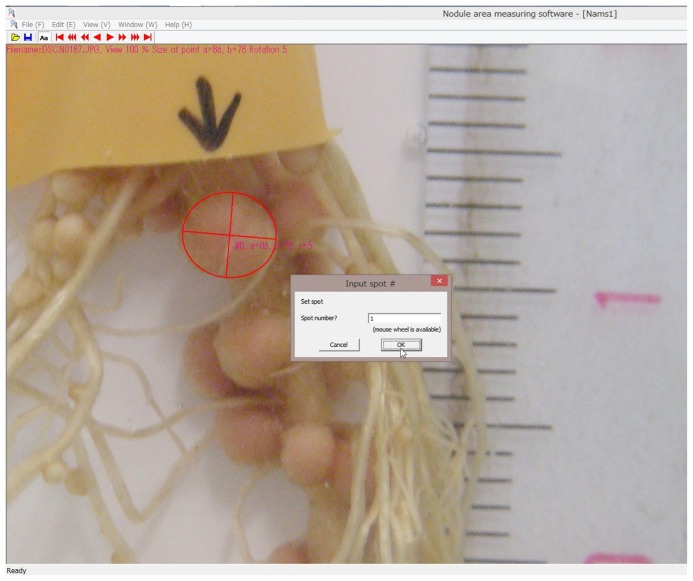
Software for measuring nodule area. A close-up photograph of a nodule is fit to an oval shape on the screen by moving and revolving the red oval line. The area of the nodule is calculated by the equation “πab”, where “a” and “b” are a half of the major and minor axes, respectively.

Young soybean nodules are globular or oval in shape. Tanabata *et al*. [33] developed a new computer program in which the shape of a close-up photograph of a nodule could be fit to an oval shape on the screen by moving and revolving the red oval line (Figure 6). The major and minor axis of the fitted oval can be used for the growth parameters. We used the area of the nodule as the growth parameter using the equation “πab”, where “a” and “b” are a half of the major and minor axes, respectively. This software “Nodame; Nodule Area Measuring Software” can be downloaded as a free software from URL http://phenotyping.image.coocan.jp/ [37]. The data file of the nodule area, major and minor axes, can be obtained with a CSV file, and used for further calculations. The area of nodules showed similar values (*R* = 0.96) when measurements with a slide caliper (Vernier scale) and this method were compared. The precision (CV%) was also almost the same between the measurements with a slide caliper (CV% = 5.09) and this method (CV% = 4.03) with 5 measurements [33]. The measurement with a slide caliper may affect the root and nodules because the measurement is done outside of the water. On the other hand, taking photographs periodically through a glass container and nutrient solution are non-invasive to the root during the measurement. The “Nodame” software can be applied to a photograph taken by a scanner [33].

### 3.4. Monitoring Root Growth by the Addition of 5 mM Nitrate under Light or Dark Conditions

The inoculation and plant cultivation in vermiculite was the same as the previous experiment monitoring nodule growth. However, different containers were used for the measurement of primary root and lateral root growth. To measure the length of the primary root, a 1 L glass cylinder was used for the plant cultivation. In order to compel the primary root to grow straight, a transparent vinyl tube with approximately 10 mm diameter was used to lead root growth. To measure the growth of lateral roots, we used glass plates that restricted the growth of lateral roots into a thin space in the glass container. To assist the direction of lateral root growth, we placed the root system between two square glass plates (10 × 10 cm) at a distance of approximately 5 mm in an 800 mL glass container. In both, plants were separated into two chambers with different light conditions 13 DAP; light or dark, the same as monitoring nodule growth. The 5 or 0 mM nitrate treatments were started at the same time. Photographs of the primary root or lateral roots were taken by a digital camera (COOL PIX S10, Nikon, Tokyo, Japan) with a high resolution (2816 × 2112 pixels) for 1-h intervals. We used a flashlight when the photograph was taken under dark conditions and covered the container and shaded the camera with aluminum foil.

### 3.5. 2D-PAGE Analysis of Nodule Protein with or without Addition of 5 mM Nitrate

Soybean plants (cv. Williams) were inoculated with *B. japonicum* strain USDA110 and cultivated in vermiculite medium with N free culture solution. The 2D-PAGE profiles of nodule proteins treated with 0 or 5 mM nitrate were compared. The 0 and 5 mM nitrate treatments were imposed for 24 days from 10 DAP, and the nodules in 34 DAP plants are collected for protein analysis. A total of 2 g of fresh nodules were extracted with 6 mL of 100 mM phosphate, ascorbate buffer (pH 7.0 by NaOH) containing 1 mM EDTA·2Na, 5 mM MgCl_2_, 10 mM dithiothreitol, and 5% insoluble polyvinylpyrrolidone. The extract was washed and dialyzed in a cold room overnight in 10 mM Tris-HCl, 1 mM EDTA buffer at pH 8.0. The sample was analyzed using the O’Farrell method. In the first dimension, separation was performed by isoelectric focusing using gels containing 8 M urea, 35 g·L^−1^ of acrylamide (acrylamide:bisacrylamide, 3.31:0.19) and 20 mL of ampholine (pH 3.5–10:pH 5–7, 1:1). An aliquot of 20 mL of the nodule extract was mixed with 60 mL of lysis buffer (8 M urea, 200 mL·L^−1^ NP-40, 10 mL·L^−1^ Ampholine pH 3.5–10, 20 mL·L^−1^ of Ampholine pH 5–7, 20 mL·L^−1^ of Ampholine pH 6–8, and 50 mL·L^−1^ of 2-mercaptoethanol, 100 mL·L^−1^ of 50% glycerol (*v*/*v*)). The protein sample was subjected to isoelectric focusing electrophoresis. After isoelectric focusing electrophoresis, the gel was subjected to second dimension SDS-PAGE. Peptides were stained with a silver staining kit (Daiichi Pure Chemicals Co., Ltd., Tokyo, Japan). 2D-PAGE was analyzed by a computer program “Polyans2D” [47]. (This software is open to access in the URL http://www.kazusa.or.jp/polyans2d/ [48]).

## 4. Conclusions

The application of combined nitrogen, especially nitrate, is known to inhibit the nodule formation, growth and nitrogen fixation activity of soybean plants. In the present study, we measured the effect of supplying 5 mM nitrate on the growth of nodules, the primary root, and lateral roots in solution culture for 16 h under light or dark conditions after an 8-h dark period. Photographs of the nodulated roots were taken by a digital camera at 1-h intervals, and the sizes of the nodules were measured with newly developed computer software. Under light conditions, nodule growth was depressed approximately at 5 h after the addition of nitrate. Under dark conditions, the nodule growth rate was almost half that under light conditions, and nodule growth was further depressed by the addition of 5 mM nitrate. The extending growth of the primary root was similar to that of nodule growth under light and dark conditions and with 0 or 5 mM nitrate treatments. On the other hand, the growth of lateral roots was promoted by the addition of 5 mM nitrate, although the growth rate under dark conditions was approximately half that under light conditions. The results of this study indicate that nodule growth is sensitively affected by nitrate within a few hours after it has been supplied, and its effects on elongation of the roots was similar in the primary roots, but opposite in the lateral roots. Plant roots elongate by cell division, differentiation and cell expansion, and root growth is primarily supported by the supply of photoassimilate from the shoot. The protein profiles of 2D-PAGE on nodules treated with 0 and 5 mM nitrate showed similar patterns, although leghemoglobin content (Lba and Lbc) was slightly decreased at 25 days after the application of 5 mM nitrate. A rapid and reversible inhibition occurred in a several hours after nitrate was supplied, and this may be attributed to a decrease in the transport of photoassimilate to the nodules and preferential use in the lateral roots.
